# Diarrhoea in neonatal piglets: a case control study on microbiological findings

**DOI:** 10.1186/s40813-018-0094-5

**Published:** 2018-09-03

**Authors:** Hanne Kongsted, Karl Pedersen, Charlotte Kristiane Hjulsager, Lars Erik Larsen, Ken Steen Pedersen, Sven Erik Jorsal, Poul Bækbo

**Affiliations:** 1 Department of Animal Science, Aarhus University, Blichers Allé 20, DK-8830 Tjele, Denmark; 2 SEGES Danish Pig Research Centre, Axeltorv 3, DK-1609 Copenhagen V, Denmark; 3 National Veterinary Institute, Technical University of Denmark, Kemitorvet, 2800 Kgs. Lyngby, Denmark

**Keywords:** Piglets, Neonatal diarrhoea, Rotavirus a, *E. coli* virulence factors, EAST1, AIDA-1, *C. difficile*, *C. perfringens* type a, Beta 2, *Enterococcus hirae*

## Abstract

**Background:**

Many factors can influence the occurrence of neonatal diarrhoea in piglets. Currently, well-known pathogens such as enterotoxigenic *Escherichia coli* and *Clostridium perfringens* type C appear to play a minor role in development of disease. Other infectious pathogens may be involved. In this study, we aimed to investigate the presence of selected infectious pathogens in neonatal piglets with clinical and pathological signs of enteric disease. The association between rotavirus A, Enterococcus hirae, Clostridium difficile and *Clostridium perfringens* type A/C and diarrhoea was investigated in a case control study on piglet level. The possible role of *E. coli* virulence factors was investigated in a multistep-procedure using herd-pools of E.coli isolates to screen for their presence.

**Results:**

Rotavirus A was detected more often in cases (25%) than in controls (6%) (*P* < 0.001). The detection rate of *Enterococcus hirae, Clostridium difficile* and *C. perfringens* type A positive for beta2 genes was the same in the two groups of piglets. *C. perfringens* type C was not detected in the study. Investigations on *E. coli* virulence factors showed a high prevalence of EAST1 toxin genes (55% of tested case piglets were positive) and AIDA-1 adhesin genes (63% of toxin positive case piglets were positive) in case piglets.

**Conclusions:**

Detection of rotavirus A was statistically significantly associated with neonatal piglet diarrhoea.

An aetiologic role of *E. coli* carrying virulence factors EAST1 and AIDA-1 needs further investigation as the study points out these two factors as possible causative factors in neonatal diarrhoea.

Detection of *E.hirae*, *C.difficile* and *C. perfringens* type A carrying beta 2 genes was not associated with neonatal piglet diarrhoea. However, the study suggested that massive overgrowth by *E. hirae* could be part of the pathogenesis in some cases of neonatal diarrhoea.

**Electronic supplementary material:**

The online version of this article (10.1186/s40813-018-0094-5) contains supplementary material, which is available to authorized users.

## Background

Many sow herds experience diarrhoea in piglets within the first week of life (neonatal diarrhoea), and it is an ongoing challenge to diagnose the underlying cause of symptoms in individual herds. In many cases, the pattern of disease indicates an infectious aetiology, but no causative agents are identified by traditional laboratory investigations. Previous examinations of neonatal diarrhoea in Danish and Swedish herds suggested that *Enterococcus *spp., rotavirus A and *E. coli* carrying EAST1 virulence genes might be of significance [1–3]. Other studies suggest that *Clostridium difficile* (*C. difficile*) and *C. perfringens* type A containing beta2 toxin gene (CPA cpb2) may be relevant to investigate further in relation to neonatal diarrhoea [4–6].

This study intended to study potential aetiologies of neonatal diarrhoea in a large number of herds throughout Denmark, focusing on specific agents previously suggested to be relevant in a North European setting. We limited our detection of agents to include rotavirus A, toxigenic *E.coli* (*E.coli* carrying genes for LT, STa, STb or EAST1), CPA cpb2, *C. perfringens* type C (CPC), *C. difficile* and *E. hirae.* Rotavirus C was not included due to earlier studies indicating a very low prevalence and no association with neonatal diarrhoea in Danish and Swedish herds [1, 7, 8]. As no PED or TGE outbreaks have been described in Denmark, coronaviruses were not investigated. In order to obtain a clear definition of a diarrhoeic case vs. a non-diarrhoeic control we used a combination of clinical and pathological examinations.

## Methods

### Enrolment of piglets

The study was performed as a case control study involving diarrhoeic and non-diarrhoeic piglets from commercial production herds. Piglets from 60 herds were selected by 23 different local practitioners (1–8 herds per practitioner) and enrolled from October 2013 to October 2014. Herd owners were asked to fill in questionnaires on vaccination schemes.

Within each herd, the local practitioners were asked to select four diarrhoeic (but otherwise apparently healthy) and two non-diarrhoeic and otherwise apparently healthy 1–5 days old piglets from different litters. Antibiotic treatment against diarrhoea was prohibited in the selected piglets, but metaphylactic antibiotic treatment on the day of birth was allowed.

### Necropsy

Piglets were euthanized by a stroke to the head and marked as diarrhoeic or non-diarrhoeic. They were packaged with cooling elements and shipped by mail to Laboratory for Pig Diseases, SEGES Pig Research Centre for necropsy on the following day. This procedure is equivalent to routine laboratory diagnostics in Denmark. Histological examination of intestinal epithelium was not an option under this setup because it requires fresh material sampled immediately after euthanasia. Necropsies were performed by experienced pathologists using a standardized scheme. The diagnosis enteritis was based upon the appearance of intestinal walls ant the consistency of colon content.

From each piglet, a section of mid-jejunum was cultured for *E. coli*. Other intestinal segments and intestinal contents were evacuated and stored at − 80 °C until further use.

### Laboratory analyses

#### *E. coli* culture and PCR

Intestinal contents were streaked onto Columbia agar plates (Oxoid) with 5% calf blood and incubated aerobically at 37 °C for 24 h. For verification of the presence of *E. coli* colonies, parallel culturing on Drigalski (in house selective and indicative medium for coliforms) was performed. When *E. coli* colonies were present, two colonies per piglet were mixed in a herd pool (containing up to twelve colonies in total, from the six piglets pr. herd) and frozen at − 80 °C in Luria-Bertani broth with 15% glycerol for subsequent PCR analyses. If present, β-haemolytic colonies were selected. Otherwise, two typical non-haemolytic colonies were selected. Subcultures of all selected colonies were frozen individually using the same procedure.

**Fig. 1:**
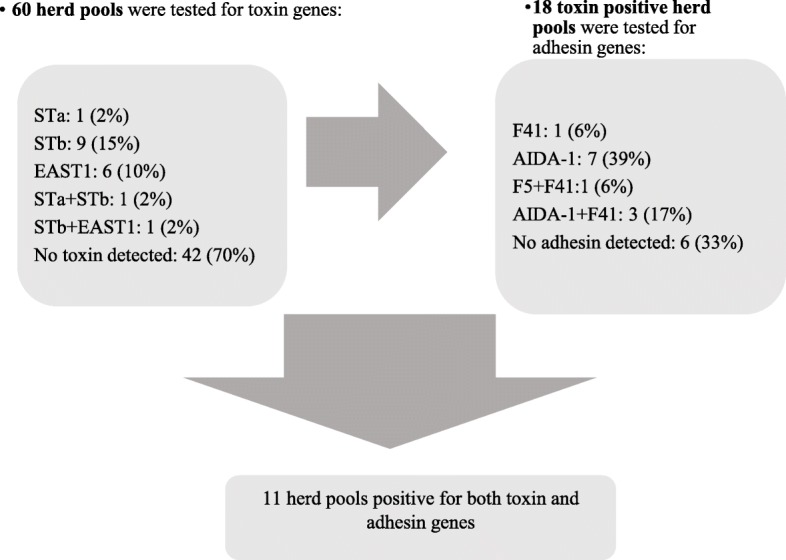
PCR results on herd pools tested for *E. coli* toxin and adhesin genes. The figure shows the number (and %) of positive pools in the stepwise procedure of PCR-testing. Note: LT, F4, F6 and F18 were not detected

PCR-analyses were performed stepwise. All herd pools (*n* = 60) were tested for the presence of heat-labile enterotoxin (LT), heat-stable toxin a (STa) and b (STb) and enteroaggregative *Escherichia coli* heat-stable enterotoxin1 (EAST1). LT, STa, and STb were detected by real-time PCR [8] in a Rotor-Gene Q real-time PCR machine (QIAGEN). Samples with Ct < 30 were considered positive. Detection of EAST1 was carried out by conventional PCR and subsequent separation by agarose gel electrophoresis. We used the method described by Zhang et al. [9] with the exception that AmpliTaq Gold Polymerase (ThermoFisher Scientific) was used and amplification was conducted in a total volume of 50 μl including 5 μl template in a TRIO thermocycler (Biometra GmbH, Germany). Herd-pools that were positive for LT, STa, STb or EAST1 were tested for the fimbrial adhesins F4, F5, F6, F18 and F41 (by real-time PCR as described for LT, STa and Stb) and adhesin involved in diffuse adherence-1 (AIDA-1) (by conventional PCR as described for EAST1). The stepwise procedure used for herd-pool testing is shown in Fig. 1.

Isolates from herd pools being positive for minimum one toxin gene and one adhesin gene were tested for STb and EAST1 genes (the most prevalent toxin genes in the herd pools). Subsequently, piglets that were positive for EAST-1 and/ or STb genes were tested for F41 and AIDA-1 genes (the most prevalent adhesin genes in the herd pools). For each virulence factor, we tested two isolates per piglet and considered a piglet positive if at least one of the two tested isolates was positive for the virulence factor in question.

#### *E. hirae* culture and identification by MALDI-TOF

A section of distal jejunum was used for culturing of *E. hirae*. Intestinal contents were plated on Slanetz-Bartley agar (Oxoid) and incubated aerobically at 37 °C for 48 h. Dominant colony types (1–3 colony types per specimen), were subcultured on blood agar and identified to species-level using MALDI-TOF (Bruker Daltronics, Bremen, Germany). Growth of *E. hirae* was semi quantitatively assessed. In the analyses we evaluated 1) Presence of *E. hirae* and 2) Massive growth of *E. hirae* (pure or massive growth).

#### PCR on intestinal contents

Jejunal contents were used for detection of rotavirus A, *C. difficile* and *C. perfringens*. Samples were prepared as a 10% suspension in PBS, beated for 20 s at 15 Hz, and centrifuged at 10.000 rpm for 90 s. Nucleic acids were extracted from 200 μl supernatant with the QIAsymphony DSP Virus/Pathogen Mini Kit (QIAGEN, Denmark), protocol “Complex 200 V6 DSP” without carrier, automated on the QIAsymphony (QIAGEN) extraction robot. Nucleic acid extracts were stored at − 80 °C until analysis.

Previously published RT-qPCR assay primers and probe targeting the NSP3 gene and designed to detect all rotavirus A genotypes from humans and animals, were used [10]. Each PCR reaction contained 2 μl template, 1 x RT-PCR Buffer and 1 x RT-PCR Enzyme Mix (AgPath-ID™ OneStep RT-PCR Kit, ThermoFisher Scientific), 400 nM of each primer and 120 nM probe in a total volume of 15 μl. PCR was performed on Rotor-Gene Q real-time PCR machine with cycling conditions: 10 min at 45 °C, 10 min at 95 °C, 48 cycles of 15 s at 95 °C, 45 s at 60 °C. Samples with Ct < 33 were considered positive.

Detection of *C. difficile* was carried out by the PCR method described by Penders et al. [11] with the exception that detection was carried out on a Rotor-Gene Q real-time PCR machine. Purified DNA from reference strain CCUG 4938 served as positive control in the PCR. Samples with Ct < 37 were considered positive.Alfa-, beta- and beta2 toxin genes related to *C. perfringens* were detected by PCR using primers and probes described previously [12]. Primers and probes were multiplexed on Rotor-Gene Q with FAM-BHQ1 fluorophore pair for the alfa-toxin gene probe, HEX-BHQ1 for the beta-toxin gene probe and Cy5-BHQ2 for the beta2-toxin gene probe. PCR reactions contained 1× JumpStart™ *Taq* ReadyMix™ (SIGMA-Aldrich), 3.5 mM MgCl_2_, 300 nM of each primer, 400 nM of each probe and 3 μl template in a total volume of 25 μl. Results for alfa-toxin, beta-toxin and beta2-toxin were detected in the green, yellow and red channels, respectively. Cut-off was Ct = 37. Only samples with positive results for alfa toxin genes were considered *C. perfringens* positive.

#### Case/control definition and statistical evaluation

A case piglet was defined as a piglet selected as diarrhoeic in the herd and given the diagnosis enteritis at necropsy. A control piglet was defined as a piglet selected as non-diarrhoeic in the herd and found healthy at necropsy. Fisher’s exact test (significance level = 0.05) was used to test statistical significant differences between microbiological findings in cases vs. controls.

## Results

### Piglets selected in the herds and herd vaccination protocols

In total, 230 piglets selected as diarrhoeic and 125 piglets selected as non-diarrhoeic were submitted from 60 herds in the study (not all herds followed the instructions on submitting four diarrhoeic and 2 non-diarrhoeic piglets). Fourtynine (82%) of the participating herds returned the questionnaires on vaccination routines. Almost all (94–98%) of these herds vaccinated breeding stock against enterotoxigenic *E. coli* (*ETEC*) and *C. perfringens* type C (mainly by using polyvalent vaccines containing *E. coli* F4, F5, F6 and *C. perfringens* betatoxoid antigens). Twenty-seven % of the herds returning questionnaires vaccinated breeding stock against *C. perfringens* type A.

#### Necropsy and inclusion as cases and controls

Extra-intestinal diagnoses were scarce. Pneumonia, arthritis, trauma from castration and unspecific findings were registered in 2, 3, 3, 9 piglets, respectively. Diagnoses at necropsy were not consistently in agreement with the clinical diagnoses in the herds. Thus, 11% of piglets with diarrhoea and 8% of the piglets selected as healthy in the herds were diagnosed with starvation as the only diagnosis at necropsy. Also, 13% of the piglets selected as diarrhoeic were diagnosed as healthy and 9% of the piglets selected as healthy were diagnosed with enteritis at necropsy (Table 1).

**Table 1:** Diagnoses assigned at necropsy in 230 piglets selected as diarrhoeic and 125 piglets selected as non-diarrhoeic by the veterinary practitioners in the herds prior to euthanazia. Single piglets were given one to two diagnoses at necropsy

	Enteritis	Starvation	Arthritis	Pneumonia	Trauma from castration	Healthy	Unspecific
Diarrhoeic	171 (74%)	31 (13%)^a^	2 (1%)	1 (0.5%)	2 (1%)	30 (13%)	4 (2%)
Non-diarrhoeic	11 (9%)	11 (9%)^b^	1 (1%)	1 (1%)	1 (1%)	97 (78%)	5 (4%)

^a^26 (11%) had starvation as the only diagnosis. ^b^10 (8%) had starvation as the only diagnosis

Altogether, 171 (74%) of the diarrhoeic piglets were diagnosed with enteritis and 97 (78%) of the non-diarrhoeic piglets were diagnosed as healthy at necropsy. These two groups of piglets served as cases and controls in the study. Descriptive information on necropsy findings in case and control piglets is given in Additional file 1.

#### Laboratory analyses

##### *E. coli*

As described, detection of *E. coli* virulence factors was performed stepwise, initially testing herd pools of *E. coli* isolates from all 60 herds submitting piglets for the study. Toxin genes were detected in 30% of herd pools with STb being the most common toxin gene. Figure 1 summarizes the stepwise testing procedure and PCR results on pool level. In total, eleven herd pools (18%) were positive for both toxin and adhesin genes.

Fourty-five piglets from these eleven herds were selected as either cases (*n* = 29) or controls (*n* = 16) and thus tested individually. More case (55%) than control piglets (19%) tested positive for *E. coli* toxin genes STb and/or EAST-1 (*P* = 0.03). Sixty-three percent of toxin gene positive piglets were positive for the adhesion gene AIDA-1, whereas F41 was not detected in any (Table 2).

**Table 2:** *E. coli* virulence factors detected in case and control piglets (only isolates from herd-pools with positive results for both toxin(s) and adhesin(s) were individually tested)

Piglets tested for EAST-1 and STb genes (*n* = 45)	Case piglets (*n* = 29)	Control piglets (*n* = 16)	*P*-value*
EAST-1	6 (21%)	1 (6%)	0.4
EAST-1 + STb	10 (34%)	2 (13%)	0.2
Toxin detected	16 (55%)	3 (19%)	0.03
Piglets tested for F41 and AIDA-1 genes (*n* = 19)	Case piglets (*n* = 16)	Control piglets (*n* = 3)	
AIDA-1^#^	10 (63%)	2 (67%)	1
F41	0 (0%)	0 (0%)	1

*Two-sided Fisher’s exact test. ^#^All piglets that were positive for AIDA-1 were positive for both EAST-1 and STb

#### *C. perfringens*, *C. difficile*, *E. hirae* and rotavirus a

CPA cpb 2 and *C. difficile* were frequently detected in both case and control piglets. Thus, more than half of the piglets in both groups were *C. difficile* positive by PCR and close to 100% of piglets in both groups were positive for CPA cpb2. None of the 268 piglets tested positive for *C. perfringens* type C (=beta toxin genes). Approximately 40% of piglets in both groups were positive for *E. hirae* by culture, but only 14% (16% of case piglets vs. 9% of control piglets (*P* = 0.1)) exhibited abundant or massive growth of this bacterial species on agar plates.

A low prevalence of Rotavirus A was detected (18% of piglets were positive by PCR), however, Rotavirus A was the only agent statistically significantly associated with case piglets (*P* = 0.001). Results on the detection of *C. difficile*, CPA cpb2, *E. hirae* and rotavirus A are presented in Table 3. Several other Streptococcus and Enterococcus species were also commonly found, most commonly *E. faecalis*, *E. faecium*, *E. durans*, *S gallolyticus* or *S. bovis.*

**Table 3:** Comparative results on *E. hirae* culture and PCR detection of rotavirus A, *C. difficile* and *C. perfringens* type A carrying beta2 genes (CpA-cpb2) in case vs. control piglets

	Case piglets (*n* = 171)	Control piglets (*n* = 97)	*P*-value*
*C. difficile*	111 (65%)	55 (57%)	0.2
CpA cpb2	157 (96%)	90 (97%)	1
*E. hirae* present	76 (44%)	42 (43%)	0.9
Massive growth of *E. hirae*	28 (16%)	9 (9%)	0.1
Rotavirus A	42 (25%)	6 (6%)	< 0.001

*Two-sided Fisher’s exact test

## Discussion

The object of this study was to investigate potential microbiological aetiologies of diarrhoea in a large number of herds throughout Denmark in order to elucidate the potential significance of specific agents.

Studies using fluorescence in situ hybridization (FISH) and multiplex qPCR (“Gut Microbiotassay”) methods previously suggested a potential significance of *Enterococcus spp*. and non-ETEC *E. coli* in Danish cases of neonatal diarrhoea [13, 14]. Furthermore, the study by Hermann-Bank et al. showed a disturbed bacterial composition of the gut flora in diarrhoeic piglets. These studies have served as an inspiration when choosing the focus of the current study. However, we chose not to include FISH and “Gut Microbiotassay” analyses in the present study, as we wanted to work under standard diagnostic conditions. We used piglets euthanized the day before necropsy and limited ourselves from using these methods.

Diarrhoeic cases and healthy controls were defined using a combination of clinical and pathological diagnostics. This approach reduced the number of clinically evaluated piglets from 355 initially submitted to 268 piglets finally included as cases and controls based on necropsy findings. The fact that 24% of piglets selected as diarrhoeic cases in the herds were either diagnosed with starvation or as being healthy at necropsy shows that diarrhoeic symptoms do not always reflect an enteric pathological condition. Furthermore, 9% of the piglets selected as healthy controls were diagnosed with enteritis at necropsy emphasizing that it is challenging to select the correct piglets for laboratory confirmation. Further, this might explain a part of the difficulties reported in relation to diagnosing causes of neonatal diarrhoea in other studies [3, 15].

Out of several infectious agents investigated, only rotavirus A was statistically significantly associated with being a case. Rotavirus A is by many considered ubiquitous in suckling piglets, but previous Danish studies have shown that the prevalence within the first week of life can be very low [16]. Studies focusing on the possible viral aetiology of the newly described phenomenon New Neonatal Porcine Diarrhoea Syndrome (NNPDS) conclude that viruses do not seem to pose a significant contribution to diarrhoeal symptoms in affected herds [7, 16]. However, these herds (four in Denmark and ten in Sweden) were selected as representative for a new type of diarrhoea not caused by known agents, including rotavirus A [1]. In the present study, we did not focus on NNPDS and did not exclude any herds due to presence of rotavirus A. Instead, we included a large number (60) of different herds in order to get a broader picture of diarrhoeal aetiologies in neonatal pigs. Interestingly, our findings support a recent study in two Danish herds. In that study, examining samples from 132 neonatal piglets, 89% of diarrhoeic piglets vs. 48% of non-diarrhoeic piglets were rotavirus A positive by PCR [2]. Taken together, these results suggest that rotavirus A has an important clinical significance in neonatal piglets, although the prevalence of case herds may be rather low as indicated by the low prevalence (18%) shown in this study.

Piglets in the study seemed to be protected against ETEC and *C. perfringens* type C by sow vaccination. The low herd-pool prevalence of *E. coli* fimbrial genes, including the previously clinically important F4 gene that was not detected at all, and the fact that *C. perfringens* type C was not detected at all probably reflects a successful vaccination scheme. Also, antibiotics used as metaphylaxis in some of the herds and the Danish breeding programme for F4 resistant pigs [17] could have an influence on the absence of F4 positive *E. coli*. STb was the only ETEC-associated toxin gene detected at a relatively high prevalence (19% of herd pools were positive). Whether a result of vaccination or not, *E. coli* results in this study support laboratory surveillance data indicating that ETEC are no longer dominant agents in neonatal diarrhoea [5, 18]. Also *C. perfringens* type C is an extremely rare agent detected in routine diagnostics in Denmark.

PCR results from *E. coli* herd pools in this study suggested that the toxin gene EAST1 and the adhesion gene AIDA-1 could be relevant to investigate further, as both these virulence factors were moderately prevalent. Few studies have evaluated the possible role of EAST1 and AIDA-1 in neonatal diarrhoea. A Danish study found that 48% of diarrhoeic vs. 18% of non-diarrhoeic neonatal piglets were positive for EAST1. In one of the investigated herds in this study, ten out of ten diarrhoeic piglets were positive for EAST1 [1].Vu-Khac et al. detected EAST1 genes in 65% of *E. coli* isolates from diarrhoeic piglets vs. 27% of isolates from non-diarrhoeic ones [19]. Other studies did not find any association between the detection of EAST1 and diarrhoea [20–22]. Previous studies on AIDA-1 found a quite low prevalence (3–17%) in isolates from diarrhoeic suckling piglets [20, 21, 23–25]. Two of these studies included non-diarrhoeic specimens and did not detect AIDA-1 in any of those [20, 21]. The current study provides limited information on the piglet level, as only 45 piglets from toxin and adhesin positive pools were individually tested as cases and controls. However, EAST1 being present in 55% of individually tested case piglets with the majority being positive for AIDA-1 also, supports relevance of further investigation of the role of these virulence factors in neonatal diarrhoea.

*E. hirae* is part of the normal intestinal flora of pigs [26], but Swedish and Danish studies have suggested a possible relation to neonatal diarrhoea [3, 13]. The Swedish study examined 29 piglets and found that 100% of diarrhoeic piglets vs. none of the non-diarrhoeic control piglets exhibited enteroadherent enterococci in the small intestine when examined by fluorescence in situ hybridisation. The Danish study examined 101 piglets and found that 37% of diarrhoeic vs. 14% of non-diarrhoeic specimens had enteroadherent enterococci in the small intestine. The present study did not find any association between the detection of *E. hirae* in intestinal contents and diarrhoea, but the results indicated that a massive growth of *E. hirae* might have clinical impact. The massive growth seen in some of the piglets in this study may, however, merely be a reflection of a disturbed microbiota.

It has been established that *C. difficile* can cause enteritis in neonatal piglets [27–29], but epidemiologic studies do not support the idea of *C. difficile* being a primary diarrhoeic pathogen in pigs [4, 13, 30–32]. In accordance with this, the detection of *C. difficile* was not associated with being a case in this study. However, it may be that histopathological examination is essential in the diagnostics on *C. difficile* related diarrhoea, and that studies merely detecting the agent or its toxins give misleading results.

Many studies have found that CPA cpb2 does not seem to be associated with neonatal diarrhoea [6, 32–34], which is supported by this study. For many years, *C. perfringens* type A has been a suspected pathogen in neonatal diarrhoea, but no studies have convincingly supported this claim. One study demonstrated a (minor) cytotoxic effect of supernatant from porcine CPA cpb2, but also showed that the cytotoxic effect was not related to beta2 toxin [35]. Thus, that study suggests that the potential role of *C. perfringens* type A in neonatal diarrhoea is not due to its ability to produce beta2 toxin. Investigations into the diarrhoea-causing capability of other toxins produced by *C. perfringens* type A may be relevant.

## Conclusions

Rotavirus A was the only agent in the study statistically significantly associated with diarrhoea, and probably plays an important role in the development of neonatal diarrhoea in some herds. The study stresses that rotavirus A is not ubiquitous in neonatal piglets. The detection of this virus in cases of neonatal diarrhoea can therefore be a useful method to support decisions on vaccination regimes.

The possible role of the *E. coli* virulence factors EAST1 and AIDA-1 needs further investigation as our results suggested these factors to be more relevant in relation to neonatal diarrhoea in today’s pig herds than the classical ETEC virulence factors.

CPC was not detected in any of 60 herds in the study and seems to be well controlled by vaccination as well as classical ETEC. Detection of *E.hirae*, *C.difficile* and CPA cpb2 was not related to diarrhoeal status. However, results in a small subset of piglets show that massive overgrowth by *E. hirae* could be part of the pathogenesis in some cases of neonatal diarrhoea.

## Additional file

Additional file 1: Necropsy findings in 171 case piglets vs. 97 control piglets. Detailed descriptive data on necropsy findings in all piglets included in the study as cases or controls. (DOCX 16 kb)
