# Supplementary material for: Diarrhoea in neonatal piglets: a case control study on microbiological findings
Source: Porcine Health Manag. 2018 Sep 3;4:17. doi: 10.1186/s40813-018-0094-5 (PMC6120089; doi:10.1186/s40813-018-0094-5)
Supplement: Supplementary file 1 — Necropsy findings in 171 case piglets vs. 97 control piglets. Detailed descriptive data on necropsy findings in all piglets included in the study as cases or controls. (DOCX 16 kb) [file 40813_2018_94_MOESM1_ESM.docx]

|  | Cases  (n=171) | Controls  (n=97) |
| --- | --- | --- |
| **External findings** |  |  |
| Male gender | 102 (60%) | 59 (61%) |
| Poor body condition | 70 (41%) | 24 (25%) |
| Wounds fore knee | 62 (36%) | 40 (41%) |
| Faecal staining | 107 (63%) | 5 (5%) |
| Dehydration^a^ | 25 (15%) | 0 (0%) |
| **Internal findings** |  |  |
| Empty^b^ stomach | 23 (13%) | 27 (28%) |
| Non visible lacteals in mesentery^c^ | 102 (62%) | 39 (41%) |
| Small intestinal flaccidity | 92 (54%) | 26 (27%) |
| Watery contents small intestine^d^ | 60 (36%) | 18 (19%) |
| Enlarged lymph nodes small intestine | 44 (26%) | 11 (11%) |
| Large intestinal flaccidity | 100 (58%) | 4 (4%) |
| Watery contents in colon^d^ | 123 (78%) | 1 (1%) |

Necropsy findings in 171 case piglets vs. 97 control piglets.

a: 24% of the dehydrated piglets had empty stomachs. b: No or almost no visible contents. c: This registration was missing from 11 piglets. 3% of piglets with non-visible lacteals had an empty small intestine. d: Piglets with empty intestinal segments were excluded from this calculation.
